# Pathogenic roles of immunoproteasomes in fibrosis

**DOI:** 10.1042/EBC20253027

**Published:** 2025-08-26

**Authors:** Sumin Kim, Seh Hoon Park, Min Jae Lee

**Affiliations:** 1 Department of Biochemistry and Molecular Biology, Seoul National University College of Medicine, Seoul, 03080, South Korea; 2 Department of Biomedical Sciences, Seoul National University Graduate School, Seoul, 03080, South Korea; 3 Department of Pathology and Laboratory Medicine, University of California, Los Angeles, Los Angeles, CA, 90095, U.S.A.; 4 Ischemic/Hypoxic Disease Institute, Convergence Research Center for Dementia, Medical Research Center, Seoul National University, Seoul, 03080, South Korea

**Keywords:** fibrosis, immunoproteasome, proteasome, ubiquitination

## Abstract

The 26S proteasome is a multi-subunit protease complex that degrades most eukaryotic cellular proteins. It not only regulates individual protein’s half-lives but also maintains proteome homeostasis and modulates immunological responses. During conditions involving large-scale proteome remodeling, such as fibrosis and cellular differentiation, the 26S proteasome plays a central role in the rapid removal of excess cytosolic proteins. However, the precise mechanisms underlying this process remain unclear. In this review, we highlight the significance of the immunoproteasome, a specialized variant of the proteasome composed of alternative catalytic subunits, in fibrosis of the kidney, lung, heart, and liver. Immunoproteasomes broaden the antigen repertoire by producing distinct peptide fragments that are preferentially presented to specific immune cell populations. They can also proteolyze substrates with certain ubiquitin (Ub) chain linkages or even those lacking Ub tags. We propose that the immunoproteasome functions as a highly specialized protease in fibrotic tissues, contributing to the transition from a complex but homeostatic proteome to a simple fibrotic proteome.

## Introduction

**Figure 1:**
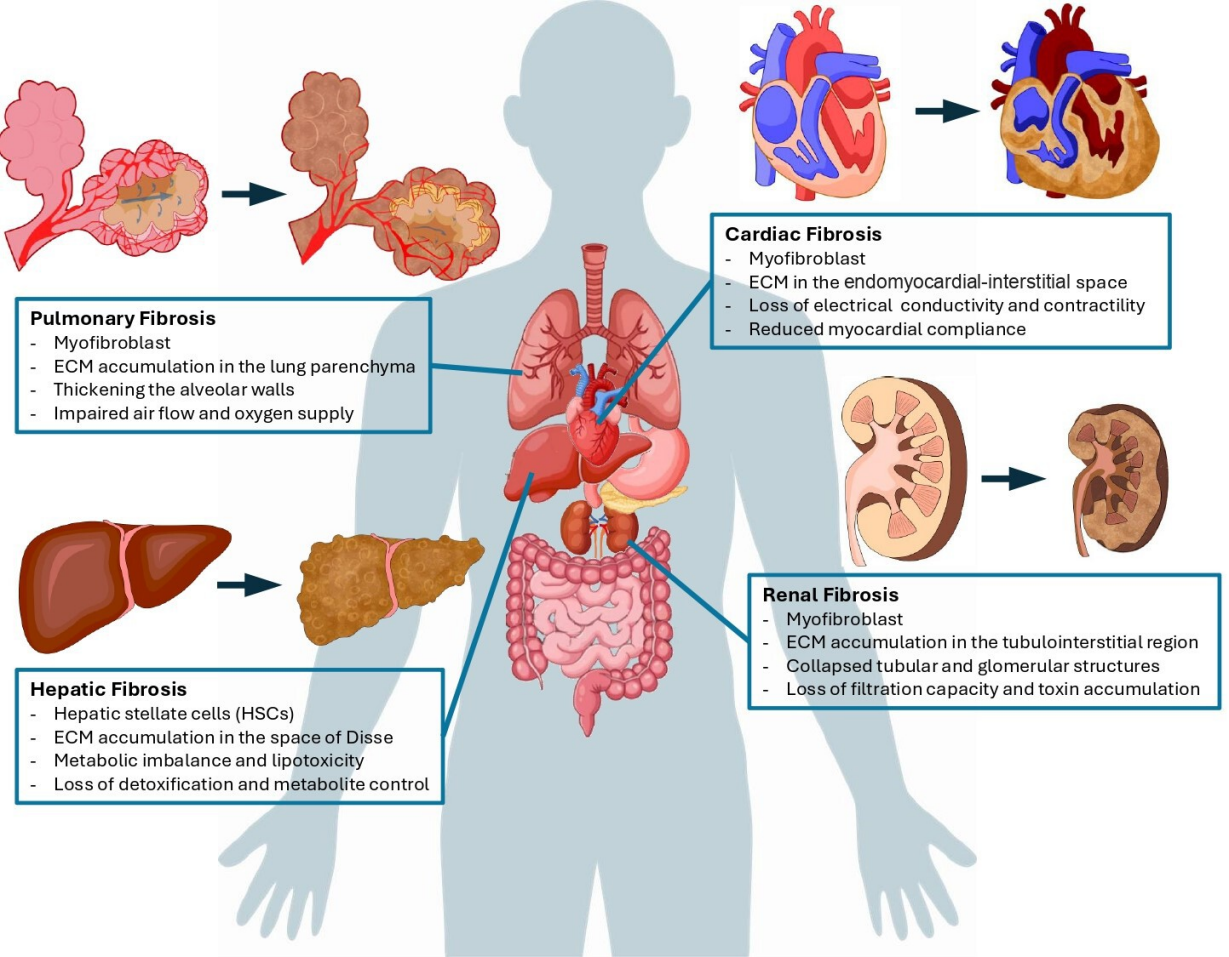
Manifestation of fibrosis in different organs. Fibrosis affects various organs and is characterized by excessive accumulation of extracellular matrix (ECM) proteins, which is primarily caused by the activation of fibroblasts or tissue-specific precursor cells. In the lung, activated myofibroblasts secrete and accumulate ECM proteins in the parenchymal area. These changes thicken the alveolar walls and disrupt alveolar architecture, impairing gas exchange. In the heart, myofibroblast-secreted ECM accumulates in the endomyocardial-interstitial space, affecting electrical conductance and contractility, and ultimately leading to reduced myocardial compliance. In the kidney, accumulated ECM components produced by activated myofibroblasts accumulate in the tubulointerstitial region and collapse tubular and glomerular structures. Consequently, the kidney’s ability to filter circulating blood is severely impaired. In the liver, HSCs and myofibroblasts form stiff ECM layers in the space of Disse, causing a metabolic imbalance and loss of detoxification capacity. These organ-specific features highlight the shared pathological markers of fibrosis, progressive tissue remodeling, and loss of function across diverse anatomical systems.

Fibrosis is a pathological condition marked by the abnormal accumulation of extracellular matrix (ECM) components, primarily collagen, resulting in tissue scarring and organ dysfunction [1]. This process is characterized by a complex interplay among tissue injury, inflammation, and defective repair processes in various organs, including the liver, lung, kidney, heart, and skin (Figure 1). While fibrosis may initially serve as a beneficial and adaptive response to acute tissue injury, its persistence can lead to irreversible organ failure, contributing to a range of diseases, including chronic kidney disease, chronic obstructive pulmonary disease (COPD), atherosclerosis, alcoholic liver disease (ALD) and non-alcoholic fatty liver disease (NAFLD), and cystic fibrosis. The severity of fibrosis is a major predictor of patient morbidity and mortality. Despite growing knowledge of fibrogenesis, there are currently no effective pharmacological interventions that can fully reverse the fibrotic process [2].

Multiple intrinsic, autocrine, and epigenetic factors trigger key fibrogenic pathways. In response to fibrogenic stimuli, resident and infiltrating immune cells release cytokines, most notably transforming growth factor β (TGFβ), that activate fibroblasts and transform them into aggressive ECM-producing cells [3]. Fibrosis also causes substantial changes in protein expression in affected tissues, which are influenced by transcriptional and epigenetic modulation. However, in recent years, the ubiquitin (Ub)-proteasome system (UPS) has emerged as a significant contributor to fibrogenesis, with the identification of numerous E3 Ub ligases involved in the pathologic process [4]. Therefore, understanding the intricate relationship between the UPS and fibrosis may provide novel insights into the pathogenesis of fibrotic disorders. This review aims to explore the current understanding of the immunoproteasome in the pathogenesis of fibrosis across various organs, with emphasis on potential therapeutic strategies. Please note that this review uses the HUGO gene names (https://www.genenames.org/).

## Ubiquitin**-p**roteasome **s**ystem and **i**mmunoproteasome

The UPS is the primary degradation system that determines the half-lives of individual proteins and controls the size and composition of the global proteome. It also removes damaged and misfolded proteins, thereby preserving protein homeostasis and cellular integrity [5]. Proteins targeted for proteasomal degradation are tagged with Ub through enzymatic processes involving E1, E2, and E3 enzymes [6]. Recent cryo-electron microscopy studies have greatly advanced our understanding of the 26S proteasome’s structure and mechanism [7,8]. The 26S proteasome is a multi-subunit holoenzyme composed of two distinct sub-complexes: the catalytic 20S core particle and the 19S regulatory particle [9,10]. The 20S proteasome has a barrel-shaped structure with seven proteasome 20S subunit alpha (PSMA)/α (α1–α7) and seven proteasome 20S subunit beta (PSMB)/β subunits (β1–β**7**) stacked in four rings (αββα). The outer PSMA ring acts as a gate for substrate entry, whereas the PSMB ring carries out proteolysis of unfolded and translocated substrates [11,12]. Only three of the seven PSMB subunits, PSMB5/β5, PSMB6/β1, and PSMB7/β2, are catalytically active, exhibiting chymotrypsin-like, caspase-like, and trypsin-like activities, respectively.

The 26S proteasome also plays a significant role in the immune response by producing antigenic peptides presented in major histocompatibility complex (MHC) class I complex [13]. Over three decades ago, scientists discovered that Interferon γ (IFNγ) stimulates the expression of additional MHC-encoded catalytic subunits, which were later identified as PSMB8/β5i, PSMB9/β1i, and PSMB10/β2i [14,15]. During *de novo* proteasome biosynthesis, these inducible PSMBi/βi subunits are assembled into the immunoproteasome, whereas the incorporation of conventional PSMB subunits is proportionally reduced [16]. Immunoproteasomes preferentially generate more peptide fragments with a hydrophobic C-terminus, which are better suited for MHC class I loading [17,18]. IFNγ-inducible proteasome activator α/β (PA28α/β) heterohexamers, in an α3/β3 stoichiometry, bind to the 20S immunoproteasome, altering its activity toward production of immunity-relevant peptides [19]. In addition, environmental exposures such as cigarette smoking, air pollution, and unhealthy diet have been shown to up-regulate immunoproteasome expression, presumably through increased pro-inflammatory cytokines and oxidative stress [20–23]. These findings raise the important question: how does the immunoproteasome function under fibrotic conditions, where immune cells and fibroblasts engage in reciprocal signaling that drives both tissue repair and pathological remodeling?

## Renal Fibrosis

Kidney fibrosis is the most prevalent pathological outcome of both acute and chronic renal disorders, and it can ultimately lead to kidney failure [24]. A variety of cell types, including fibroblasts, pericytes, endothelial cells, and tubular epithelial cells, can differentiate into active myofibroblasts, which secrete ECM proteins such as collagen and fibronectin. Excessive ECM accumulation results in the formation of a dense, rigid fibrotic matrix inside the tubulointerstitial space, causing collapse of glomeruli and vascular structures and disrupting overall renal architecture and function [25]. Once fibrosis progresses, its regenerative capacity is greatly diminished, frequently leading to irreversible progression to end-stage renal disease, which requires dialysis or kidney transplantation. Clinical markers such as estimated glomerular filtration rate (eGFR) typically reveal damage only after substantial functional deterioration has occurred. Therefore, establishing early diagnostic techniques is critical for enabling interventions before renal repair mechanisms are permanently impaired in progressive kidney diseases.

Over the past decade, the role of ubiquitination in renal fibrosis has received considerable attention. Several E3 Ub ligases and deubiquitinating (DUB) enzymes have been linked to fibrotic signaling pathways, including TGFβ, Wnt, mitophagy, inflammation, and oxidative stress [26,27]. SMAD specific E3 ubiquitin-protein ligase 2 (SMURF2) and Arkadia promote the ubiquitination and degradation of SMAD family member 7 (SMAD7), leading to enhanced TGFβ signaling and fibrogenesis [28,29] (Table 1). Other E3 Ub ligases, including Parkin RBR E3 ubiquitin protein ligase (PRKN), Tripartite motif containing 31 (TRIM31), and HECT, UBA, and WWE domain containing E3 ubiquitin protein ligase 1, contribute to fibrotic progression by modulating mitophagy, inflammatory responses, and the Janus kinase (JAK)-signal transducer and activator of transcription (STAT) pathway [30–34]. Synoviolin 1 (SYVN1) and neural precursor cell expressed developmentally down-regulated 4 (NEDD4L) regulate the β-catenin complex and Wnt signaling through distinct mechanisms that also promote fibrosis [35,36]. However, it should be noted that the direct mechanistic connection between these E3 ligases and immunoproteasomes remains insufficiently understood to draw a definitive conclusion. DUB enzymes, such as Ubiquitin specific peptidase 4 (USP4) and USP11, enhance TGFβ signaling by deubiquitinating TGFβ receptors, while USP25 stabilizes SMAD4 and prevents SMAD2 nuclear translocation, thereby sustaining downstream TGFβ signaling [55–57].

**Table 1:** Ubiquitin ligases that are involved in the progression or inhibition of organ fibrosis

Organ	E3 ubiquitin ligase	Substrate	Related pathway	Fibrotic effect	Reference
Kidney	SMURF1/2	SMAD7, SKIL, and SKI	TGFβ	Profibrotic	[28]
	Arkadia	SMAD7	TGFβ	Profibrotic	[29]
	PRKN	MFN2	Mitophagy	Antifibrotic	[30]
	TRIM31	MAP3K7	MAPK and TGFβ	Profibrotic	[31]
	TRIM39	PRDX3	Oxidative stress	Profibrotic	[32]
	TRIM65	NUDT21	TGFβ	Profibrotic	[33]
	HUWE1	EGFR	-	Antifibrotic	[34]
	SYVN1	GSK3B and CSNK1A1	Wnt/β-catenin and TGFβ	Profibrotic	[35]
	NEDD4L	DVL2	Wnt/β-catenin	Antifibrotic	[36]
Lung	SMURF2	SMAD7 and HSPB1	TGFβ	Profibrotic	[37]
	NEDD4	SMAD2/3, TGFβRII and YY1	TGFβ	Antifibrotic	[38] [39] [40]
	STUB1	NOX4	Oxidative stress	Antifibrotic	[41]
	FIEL1	PIAS4	TGFβ	Profibrotic	[42]
Heart	TRIM72	-	STAT3/NOTCH1	Profibrotic	[43]
	TRIM21	-	PI3K/AKT serine/threonine kinase (AKT)	Profibrotic	[44]
	WWP2	PARP1	-	Antifibrotic	[45]
	PELI1	-	TGFβ	Profibrotic	[46]
	SPOP	RACK1	TGFβ	Profibrotic	[47]
	FBXL8	SNAI1	TGFβ and RHOA/α-SMA	Antifibrotic	[48]
Liver	STUB1 and NEDD4	PPARγ	Metabolic regulation	Antifibrotic	[49,50]
	SYVN1 and PRKN	-	Mitophagy	Antifibrotic	[51,52]
	SMURF2	PDE4B	cAMP/PKA	Antifibrotic	[53]
	BFAR	PNPLA3	Cholesterol synthesis	Antifibrotic	[54]

SMURF, SMAD specific E3 ubiquitin-protein ligase. PRKN, Parkin RBR E3 ubiquitin protein ligase. TRIM, tripartite motif containing. SMAD, SMAD family member. MAP3K7, mitogen-activated protein kinase kinase kinase 7. PRDX3, Peroxiredoxin 3. TGFβ, transforming growth factor β. MAPK, mitogen-activated protein kinase. HUWE1, HECT, UBA, and WWE domain containing E3 ubiquitin protein ligase 1. SYVN1, synoviolin 1. NEDD4, neural precursor cell expressed developmentally down-regulated 4. NUDT21, nudix hydrolase 21. GSK3B, glycogen synthase kinase 3 β. CSNK1A1, Casein kinase 1 α 1. DVL2, dishevelled segment polarity protein 2. STUB1, STIP1 homology and U-box containing protein 1. FIEL1, fibrosis-inducing E3 ligase. WWP2, WW domain containing E3 ubiquitin protein ligase 2. PELI1, Pellino E3 ubiquitin-protein ligase 1. NOX4, NADPH oxidase 4. PIAS4, protein inhibitor of activated STAT 4. PARP1, Poly (ADP-ribose) polymerase 1. STAT, signal transducer and activator of transcription. NOTCH, Notch receptor 1. PI3K, phosphoinositide 3-kinase. EGFR, epidermal growth factor receptor. TGFβRII, TGFβ receptor II. SPOP, Speckle-type BTB/POZ protein. FBXL8, F-box and leucine rich repeat protein 8. SNAI, Snail family transcriptional repressor 1. PPARγ, peroxisome proliferator-activated receptor γ. PDE4B, phosphodiesterase 4B. RHOA, Ras homolog family member A. cAMP, cyclic adenosine monophosphate. PKA, protein kinase A. BFAR, bifunctional apoptosis regulator. PNPLA3, Patatin like domain 3, 1-acylglycerol-3-phosphate O-acyltransferase. α-SMA, α smooth muscle actin. HSPB1, heat shock protein family B (small) member 1. SKI, Sloan-Kettering Institute proto-oncogene. SKIL, SKI-like oncogene. YY1, Yin yang 1 transcription factor.

In addition to Ub-related modulation, recent studies have shown that immunoproteasomes participate in pathologic protein degradation during fibrogenesis. In kidney transplantation, alloantibody-mediated rejection, a major cause of chronic graft nephropathy, is associated with up-regulation of immunoproteasome activity in the kidney, spleen, and bone marrow. Post-transplantation treatment with the immunoproteasome-selective inhibitor ONX-0914 or the pan-proteasome inhibitor bortezomib has been demonstrated to reduce chronic rejection, including interstitial fibrosis [58]. These inhibitors suppress plasma cell populations by inducing severe endoplasmic reticulum (ER) stress, activating the unfolded protein response [59]. As plasma cells rely heavily on proteasome activity for antibody synthesis and secretion, dual inhibition of both constitutive and immunoproteasomes effectively reduces transplant rejection and improves recipient survival. An independent study modeling cold ischemia in kidney transplantation reported the induction of immunoproteasome subunit PSMB8 after surgery. However, ONX-0914 administration during cold storage did not alleviate fibrosis, suggesting that immunoproteasome activation is primarily driven by host immune responses rather than by the cold storage process itself [60].

Tubulointerstitial fibrosis is a common complication of immunoglobulin A nephropathy (IgAN), a chronic autoimmune glomerular disease characterized by IgA deposition in the mesangium. Patients with IgAN exhibit elevated expression of PSMB8 in peripheral blood mononuclear cells, which correlates with clinical indicators such as proteinuria and eGFR [61,62]. The presence of the immunoproteasome in this autoimmune context suggests that it could be a therapeutic target for IgAN. Furthermore, bortezomib has been shown to reduce fibrogenic activity in a murine model of aristolochic acid-induced kidney fibrosis, supporting the hypothesis that proteasome hyperactivity contributes to renal pathology [63]. In conclusion, immunoproteasomes, probably along with conventional proteasomes, play a pro-fibrotic role in the kidney by promoting tubulointerstitial fibrosis and contributing to the progression of renal dysfunction.

## Pulmonary Fibrosis

Idiopathic pulmonary fibrosis (IPF) is also characterized by the abnormal accumulation of ECM components, particularly in the lung interstitium and parenchyma, and is caused by the uncontrolled proliferation of myofibroblasts. This causes a considerable disruption to the alveolar architecture [64]. Fibrosis increases lung stiffness, reduces elasticity, and impairs gas exchange, all of which contribute to progressive respiratory dysfunction. As a common complication of chronic respiratory diseases, fibrosis exacerbates disease progression and may ultimately lead to respiratory failure.

Several E3 Ub ligases have been shown to modulate the TGFβ signaling pathway in lung fibrogenesis. For instance, SMURF2, a really interesting new gene (RING) finger E3 ligase, ubiquitinates and degrades phosphorylated heat shock protein family B (small) member 1 and SMAD7, leading to increased TGFβ signaling and lung fibrosis [37,65]. Conversely, the homologous to the E6-AP carboxyl terminus (HECT)-type E3 ligase NEDD4 targets activated SMAD2/3 and TGFβ receptor II (TGFβRII), thereby inhibiting downstream TGFβ signaling and restricting fibrotic development [38–40]. STIP1 homology and U-box containing protein 1 (STUB1)/C-terminus of HSC70-interacting protein (CHIP) ubiquitinates NADPH oxidase 4, contributing to lower oxidative stress and fibrosis, while fibrosis-inducing E3 ligase 1 targets protein inhibitor of activated STAT 4 to stimulate TGFβ signaling [41,42]. DUB enzymes such as USP11 and Ub C-terminal hydrolase L5 (UCHL5) are up-regulated in fibrotic lung tissue, where they stabilize TGFβRII and SMAD2/3, further contributing to pulmonary fibrosis [66,67]. In addition to the direct regulation of the TGFβ pathway, several other E3s and DUBs, including mouse double minute 2, BRCA1 associated RING domain 1 (BARD1), tripartite motif containing 33 (TRIM33), and USP13, have also been implicated in lung fibrogenesis [68–71].

Beyond specific signaling pathways, the 26S proteasome undergoes significant changes from the conventional to immunoproteasomes during fibrosis. Under normal physiological conditions, immunoproteasomes can be found within the lungs in interstitial immune cells, such as alveolar macrophages [72]. However, in IPF lungs, the immunoproteasome subunit PSMB9 is significantly up-regulated in epithelial cells and fibroblasts. In damaged lungs, the release of mitochondrial DNA activates the cyclic GMP-AMP synthase/stimulator of interferon response cGAMP interactor 1 (cGAS/STING1) pathway and elevates IFNβ signaling, thereby increasing immunoproteasome expression in surrounding cells.

Contradictory observations have been reported in patients with COPD and IPF, who show reduced immunoproteasome expression and broader immunological dysfunction. Both groups show low immunoproteasome levels and markedly decreased immunological activity [20]. In mouse models, cigarette smoking initially induces the expression of immunoproteasome and MHC class I-associated genes; however, chronic exposure leads to a decline in these responses, illustrating the long-term impact of pulmonary damage. Immunoproteasomes also modulate immune cell dynamics in IPF. During fibrosis, alveolar macrophages first adopt a pro-inflammatory M1 phenotype before transitioning to an M2 phenotype, which promotes tissue remodeling and wound healing—both key features of fibrotic progression [73].

Inhibiting immunoproteasomes, either by genetic deletion of PSMB8 or pharmacological treatment with ONX-0914, significantly affects the differentiation and functional capabilities of M2 macrophages despite their prevalence in fibrotic tissues. These findings align with elevated M2 macrophage levels in patients with IPF, suggesting that immunoproteasomes may help sustain a pro-fibrotic microenvironment. Furthermore, ONX-0914 has been shown to suppress macrophage differentiation in lipopolysaccharide (LPS)- and elastase-induced emphysema models [74]. A novel PSMB8-selective inhibitor reduced fibrosis in TGFβ-stimulated lung fibroblasts and bleomycin-induced IPF mouse models, reinforcing the pro-fibrotic role of the immunoproteasome [75]. Viral infections caused by MHV-68 and MHV-1—both associated with pulmonary fibrosis—also induced immunoproteasome expression [76,77]. To summarize, in pulmonary fibrosis, immunoproteasomes replace conventional proteasomes and alter proteolytic activity. They influence macrophage polarization, antigen presentation, and immune cell recruitment—processes that collectively contribute to fibrotic development and pulmonary dysfunction.

## Cardiac Fibrosis

Cardiac fibrosis, particularly endomyocardial fibrosis, is characterized by excessive ECM deposition in the heart, leading to myocardial stiffness, impaired electrical conduction, and reduced contractility [78]. This fibrotic remodeling compromises both electrical signaling and mechanical function of the myocardium and is associated with a variety of cardiac disorders, including myocardial infarction and cardiomyopathy, ultimately leading to functional heart failure. During fibrosis, resident fibroblasts differentiate into myofibroblasts, which increase the production and secretion of fibrillar collagens and other ECM proteins, thickening interstitial ECM layers [79]. Additionally, endothelial cells and cardiomyocytes can transdifferentiate into myofibroblasts, further exacerbating the disease process.

Several E3 Ub ligases and DUB enzymes play important roles in modulating cardiac fibrotic signaling. For example, TRIM72 stimulates pro-fibrogenic STAT3/Notch receptor 1 signaling, promoting myofibroblast formation, while TRIM21 promotes macrophage polarization, worsening fibrotic outcomes [43,44]. WW domain containing E3 ubiquitin protein ligase 2 (WWP2) in the myocardium ubiquitinates poly(ADP-ribose) polymerase 1, leading to cardiac remodeling, and Pellino E3 ubiquitin-protein ligase 1 augments TGFβ transcription, stimulating myofibroblast activation and fibrosis progression [45,46]. As Speckle-type BTB/POZ protein promotes fibrosis by targeting receptor for activated C kinase 1 (RACK1) to activate TGFβ/SMAD3 signaling, F-box and leucine-rich repeat protein 8 degrades Snail family transcriptional repressor 1 and suppresses myofibroblast differentiation [47,48]. Meanwhile, DUB enzymes, including USP18, USP4, USP2, USP10, and USP15, exhibit target- and context-dependent effects, acting as both pro- and anti-fibrotic regulators.

Angiotensin-infused mouse models are widely used to study cardiac fibrosis, as angiotensin exerts vasopressor effects that cause structural heart damage. This model up-regulates immunoproteasome subunits PSMB8 and PSMB10 and alters proteolytic activity [80,81]. Patients with atrial fibrosis show elevated levels of PSMB8 and PSMB10 in cardiac tissues, along with increased proteasome activity in plasma. Notably, genetic or pharmacological inhibition of immunoproteasomes stabilizes angiotensin II receptor associated protein, a cofactor of angiotensin II receptor type 1 (AGT1R), thereby suppressing AGT1R function [52,82]. This inhibition reduced angiotensin recognition and downstream fibrotic signaling. Loss of PSMB8 in angiotensin-treated neonatal rat cardiomyocytes reduces fibrosis, whereas PSMB8 overexpression enhances AGT1R signaling and TGFβ activation in cultured cells [83]. PSMB8 can also interact with ATG5, promoting its degradation and reducing autophagic flux. The pro-fibrotic effects of PSMB8 in the heart were also observed in the transverse aortic constriction mouse model, suggesting that dysregulated autophagy is a common mechanism in cardiac fibrosis along with immunoproteasome induction.

Duchenne muscular dystrophy (DMD) is a progressive myopathic condition caused by dystrophin gene mutation and is linked to cardiac fibrosis and arrhythmias. In DMD mouse models, immunoproteasome levels were elevated in cardiomyocytes. Cardiomyocytes from patients with DMD and patient-derived fibroblasts both showed similar increases in immunoproteasome levels. Treatment with ONX-0914 reduces immunoproteasome levels, improves sarcoplasmic reticulum function, decreases inflammation, and slows fibrosis progression in these models [84]. The deoxycorticosterone acetate (DOCA) model, which involves high salt intake, induces hypertension and cardiac fibrosis independently of angiotensin. DOCA-salt mice exhibit increased immunoproteasome expression, contributing to pathological hypertension and fibrosis. Genetic deletion of PSMB8 or treatment with ONX-0914 alleviated these effects [85]. Taken together, these findings show that immunoproteasomes are persistently up-regulated in fibrotic heart tissue and contribute to disease progression through multiple mechanisms, including AGT1R signaling, autophagy suppression, and immune modulation. Therefore, targeting immunoproteasomes may offer promising therapeutic benefits in various forms of cardiac fibrosis.

## Hepatic Fibrosis

Liver fibrosis, characterized by the deposition of collagen layers in the spaces of Disse, is one of the earliest and most common manifestations of chronic liver disease [86]. Hepatotoxic insults, including viral hepatitis, ALD, NAFLD, autoimmune hepatitis, and cholestatic disorders, are the primary causes of fibrosis development. Excessive fibrosis leads to liver stiffness and portal hypertension, which impairs systemic metabolism and detoxification functions. Prolonged fibrosis can progress to cirrhosis and hepatocellular carcinoma. Hepatic stellate cells (HSCs) and myofibroblasts are the primary mediators of this process, with HSCs transitioning from a quiescent to an active state and contributing significantly to fibrotic matrix deposition [87]. These cells are derived from fibroblasts and pericytes in the injured liver.

Unlike other organ-specific fibrotic processes, liver fibrosis is more closely associated with metabolic dysregulation than with TGFβ hyperactivation. E3 Ub ligases STUB1 and NEDD4 regulate hepatic homeostasis by interacting with peroxisome proliferator-activated receptor gamma (PPARγ), a nuclear receptor that controls lipid and adipocyte metabolism [49,50]. SYVN1 and PRKN, two key regulators of ER stress and mitophagy, also contribute to the progression of liver fibrosis [51,53]. Bifunctional apoptosis regulator targets Patatin like domain 3, 1-acylglycerol-3-phosphate O-acyltransferase, thereby regulating hepatic lipid metabolism [54]. Other E3 ligases and DUB enzymes are up-regulated during HSC activation, underscoring the importance and complexity of the UPS-mediated proteome remodeling in hepatic fibrosis.

Immunoproteasomes are substantially up-regulated in liver endothelial cells during the active phase of fibrosis, leading to cirrhosis in both human patients and mouse models of non-alcoholic steatohepatitis induced by carbon tetrachloride (CCl_4_) or a high-fat diet [22]. ONX-0914 has been shown to reduce fibrotic disease in mouse models and human umbilical vein endothelial cells. Given the importance of endothelial cells in immunomodulation, increased immunoproteasome expression likely enhances antigen presentation and immune cell recruitment in fibrotic livers [88]. In addition to ONX-0914, pan-proteasome inhibitors also disrupted HSC function and delayed fibrotic progression [89,90].

Substantial infiltration of immune cells during hepatic injury is involved in the transition from conventional to immunoproteasomes. During fibrosis, liver macrophage populations increase markedly, initially skewing toward a pro-inflammatory M1 phenotype before transitioning to the pro-fibrotic M2 phenotype [91]. Immunoproteasomes, present in both M1 and M2 macrophages, play crucial roles in their activation and function. Furthermore, an altered antigen repertoire in immunoproteasome-expressing hepatic cells may influence interactions with plasma cells and modulate immune responses in patients with fibrosis [92]. To summarize, immunoproteasomes are important mediators of liver fibrosis, shaping both cellular behavior and immune dynamics. Their up-regulation suggests a role in exacerbating fibrosis through immunological modulation, while their inhibition represents a promising therapeutic strategy. Future research is needed to clarify their mechanistic roles and to evaluate targeted treatments for liver fibrosis.

## Conclusions and Therapeutic Perspectives

Anti-fibrotic therapies, both licensed and under development, primarily target signaling pathways such as TGFβ, receptor tyrosine kinases, and JAKs. While these pathways play important roles in fibrotic progression, their broad engagement in diverse physiological processes may limit therapeutic specificity and constrain dosing strategies due to potential off-target effects and safety concerns. Although organ-specific metabolic regulators, such as PPARs, Farnesoid X receptor, and glucagon-like peptide-1 agonists, are being studied, they may also lack specificity for fibrotic mechanisms. Given the heterogeneous nature of the fibrotic microenvironment, combination therapies appear to be a preferred approach, underscoring the urgent need for innovative and targeted treatments.

Bortezomib (Velcade), carfilzomib (Kyprolis), and ixazomib (Ninlaro) are three FDA-approved proteasome inhibitors that suppress both conventional and immunoproteasome activity. Preclinical studies have demonstrated their ability to reduce fibrosis in the kidney, lung, and liver [63,89,90,93–97]. However, clinical investigations in patients with myeloma have revealed adverse effects on these organs [98–101]. These findings highlight the challenges associated with non-selective proteasome inhibition, which likely impairs normal protein turnover and cellular homeostasis, triggering global apoptosis. ONX-0914 (PR-957), a selective inhibitor of PSMB8, has shown promising anti-fibrotic effects across multiple organ systems, including the kidney, lung, heart, and liver (Table 2) [22,58,59,74,77,84,85]. Targeting immunoproteasomes, which are predominantly expressed in inflammatory and fibrotic tissues, offers the potential for more precise and safer therapeutic strategies. Zetomipzomib (KZR-616), a derivative of ONX-0914, is currently undergoing clinical trials for autoimmune diseases and has been rigorously evaluated for safety and efficacy. Despite this progress, challenges still remain, including concerns about off-target effects, long-term safety in heterogeneous fibrotic disorders, limited translatability from preclinical models, and a lack of robust clinical benefits for fibrosis. Nevertheless, further development and continuing clinical evaluation, particularly in combination with established therapies, are expected to establish immunoproteasome inhibitors as a novel approach for managing fibrosis.

**Table 2:** Effects of the conventional proteasome inhibitors (bortezomib and carfilzomib) and the immunoproteasome inhibitor (ONX-0914) in fibrosis

Organ	Drug	Species	Model	Drug administration				Fibrotic effect	Ref
				Route	Dose	Frequency	Duration		
Kidney	ONX-0914	Rat (F344)	Allograft	SC	5 mg/kg	2 times /wk	7 wk	Reduced	[59]
	Bortezomib	Rat (F344)	Allograft	IV	0.2 mg/kg	2 times /wk	7 wk	Reduced	[59]
		Rat (F344)	Allograft	IV	0.2 mg/kg	1 time /3 days	~16 wk	Reduced	[95]
		Mouse (C57BL/6J)	AAN	IP	0.5 mg/kg	2 times /wk	10 wk	Reduced	[63]
Lung	ONX-0914	Mouse (C3H/HeJ)	MHV-1	SC	5 mg/kg	Every day	~8 days	aggravated	[77]
		Mouse (C57BL/6J)	Emphysema (LPS+elastase)	IN	5 mg/kg	3 times /wk	4 wk	Reduced	[74]
	Bortezomib	Mouse (C57BL/6J)	Bleomycin (IT, 2.5 U/kg)	IP	0.25 mg/kg	1 time /3 days	13 days	Reduced	[96]
		Mouse (C57BL/6J)	Bleomycin (OPA, 1.5 U/kg)	IP	0.1, 0.25 mg/kg	1 time /3 days	13 days	Reduced	[93]
		Mouse (C57BL/6J)	Bleomycin (IT, 0.075 IU)	IP	120 μg/kg	1 time /wk	2 wk	Reduced	[97]
Heart	ONX-0914	Mouse (C57BL/6J)	Dystrophin-def *mdx* mouse	IP	6 mg/kg	2 times /wk	5 wk	Reduced	[84]
		Mouse (C57BL/6J)	DOCA-salt	IP	12 mg/kg	Every day	3 wk	Reduced	[85]
Liver	ONX-0914	Mouse (C57BL/6J)	CCl₄ and CCl₄ with WD	IP	10 mg/kg	1 time /3 days	3 wk	Reduced	[22]
	Carfilzomib	Mouse (C57BL/6)	CCl₄	IV	5 mg/kg	2 times /wk	5 wk	Reduced	[89]

AAN, aristolochic acid nephropathy. IP, intraperitoneal injection. IT, intratracheal injection. IV, intravenous injection. OPA, oropharyngeal aspiration. SC, subcutaneous injection. WD, Western diet. LPS, lipopolysaccharide. DOCA, deoxycorticosterone acetate. CCl_4_, carbon tetrachloride. IN, intranasal injection.

Fibrosis is a complex pathological condition associated with a wide variety of disorders. Its complexity stems from differences in organ characteristics, cellular composition, and comorbid conditions, all of which confound mechanistic understanding and therapeutic advancement. Nevertheless, one emerging theme is the consistent involvement of the immunoproteasome in different fibrotic disorders. The immunoproteasome is a unique proteolytic entity that is activated under specific pathological conditions, generating a distinct peptide pool that reshapes the antigenic landscape of damaged tissues, potentially enhancing immune cell recruitment and contributing to the chronicity of fibrotic responses. Despite many challenges, immunoproteasome-targeted approaches represent a viable path forward in anti-fibrotic therapy. The immunoproteasome also has the potential to serve as both a therapeutic target and a biomarker for fibrotic progression. While previous research focused primarily on its role in immunological and inflammatory conditions, growing evidence supports its involvement in tissue remodeling and fibrosis. Continued research is needed to fully elucidate the role of the immunoproteasome in fibrotic disease and maximize its therapeutic potential.

**Summary**

- Fibrosis is a complex pathological condition characterized by excessive deposition of the proteinaceous components of extracellular matrix .
- The immunoproteasome, a specialized and inducible variant of the proteasome, is prominently formed in various organs undergoing fibrosis, such as the kidney, lung, heart, and liver.
- The immunoproteasome influences the dynamic remodeling of the proteome and antigen presentation, thus playing a substantial role in the fibrotic pathogenesis.
- As conventional proteasome inhibitors have been associated with adverse effects, selectively targeting the immunoproteasome may offer a more precise and effective strategy for treating fibrosis.

## Abbreviations

AAN: Aristolochic acid nephropathy
AGT1R: Angiotensin II receptor type 1
AGTRAP: Angiotensin II receptor associated protein
ALD: Alcoholic liver disease
BARD1: BRCA1 associated RING domain 1
BFAR: Bifunctional apoptosis regulator
COPD: Chronic obstructive pulmonary disease
CSNK1A1: Casein kinase 1 alpha 1
DMD: Duchenne muscular dystrophy
DUB: Deubiquitinating enzyme
DVL2: Dishevelled segment polarity protein 2
ECM: Extracellular matrix
EGFR: Epidermal growth factor receptor
ER: Endoplasmic reticulum
FBXL8: F-box and leucine rich repeat protein 8
FIEL1: Fibrosis-inducing E3 ligase 1
FXR: Farnesoid X receptor
GLP-1: Glucagon-like peptide-1
GSK3B: Glycogen synthase kinase 3 beta
HSC: Hepatic stellate cell
HSPB1: Heat shock protein family B (small) member 1
HUWE1: HECT, UBA and WWE domain containing E3 ubiquitin protein ligase 1
IFNγ: Interferon gamma
IgAN: Immunoglobulin A nephropathy
JAK: Janus kinase
LPS: Lipopolysaccharide
MAPK: Mitogen-activated protein kinase
MAP3K7: Mitogen-activated protein kinase kinase kinase 7
MAPK: Mitogen-activated protein kinase
MDM2: Mouse double minute 2
NAFLD: Non-alcoholic fatty liver disease
NEDD4: Neural precursor cell expressed developmentally down-regulated 4
NOTCH1: Notch receptor 1
NOX4: NADPH oxidase 4
NUDT21: Nudix hydrolase 21
PARP1: Poly (ADP-ribose) polymerase 1
PDE4B: Phosphodiesterase 4B
PELI1: Pellino E3 ubiquitin-protein ligase 1
PIAS4: Protein inhibitor of activated STAT 4
PI3K: Phosphoinositide 3-kinase
PKA: Protein kinase A
PNPLA3: Patatin like domain 3, 1-acylglycerol-3-phosphate O-acyltransferase
PPARγ: Peroxisome proliferator-activated receptor gamma
PRDX3: Peroxiredoxin 3
PRKN: Parkin RBR E3 ubiquitin protein ligase
PTEN: Phosphatase and tensin homolog
RHOA: Ras homolog family member A
RTK: Receptor tyrosine kinase
SKI: Sloan-Kettering institute proto-oncogene
SKIL: SKI-like oncogene
SMAD: SMAD family member
SMURF: SMAD specific E3 ubiquitin-protein ligase
SNAI1: Snail family transcriptional repressor 1
SPOP: Speckle-type BTB/POZ protein
STAT: Signal transducer and activator of transcription
STUB1: STIP1 homology and U-box containing protein 1
SYVN1: Synoviolin 1
TAC: Transverse aortic constriction
TGFβ: Transforming growth factor beta
TGFβRII: TGFβ Receptor II
TRIM: Tripartite motif containing
UPS: Ubiquitin-proteasome system
USP: Ubiquitin specific peptidase
Ub: Ubiquitin
WWP2: WW domain containing E3 ubiquitin protein ligase 2
YY1: Yin yang 1 transcription factor
cAMP: Cyclic adenosine monophosphate
eGFR: Estimated glomerular filtration rate
α-SMA: Alpha smooth muscle actin
